# Using the pea aphid *Acrythociphon pisum *as a tool for screening biological responses to chemicals and drugs

**DOI:** 10.1186/1756-0500-2-185

**Published:** 2009-09-16

**Authors:** Aviv Dombrovsky, Terence Neil Ledger, Gilbert Engler, Alain Robichon

**Affiliations:** 1 Agrobiotech, INRA/CNRS/UNSA, Sophia Antipolis, France; 2 Department of Plant Protection, The Volcani Center, Bet-Dagan, Israel

## Abstract

**Background:**

Though the biological process of aphid feeding is well documented, no one to date has sought to apply it as a tool to screen the biological responses to chemicals and drugs, in ecotoxicology, genotoxicology and/or for interactions in the cascade of sequential molecular events of embryogenesis. Parthenogenetic insect species present the advantage of an anatomical system composed of multiple germarium/ovarioles in the same mother with all the intermediate maturation stages of embryos from oocyte to first instar larva birth. This could be used as an interesting model to visualize at which step drugs interact with the cell signalling pathway during the ordered developmental process.

**Findings:**

We designed a simple test for screening drugs by investigating simultaneously zygote mitotic division, the progression of embryo development, cell differentiation at early developmental stages and finally organogenesis and population growth rate. We aimed to analyze the toxicology effects of compounds and/or their interference on cellular signalling by examining at which step of the cascade, from zygote to mature embryo, the developmental process is perturbed. We reasoned that a parthenogenetic founder insect, in which the ovarioles shelter numerous embryos at different developmental stages, would allow us to precisely pinpoint the step of embryogenesis in which chemicals act through specific molecular targets as the known ordered homeobox genes.

**Conclusion:**

Using this method we report the results of a genotoxicological and demographic analysis of three compound models bearing in common a bromo group: one is integrated as a base analog in DNA synthesis, two others activate permanently kinases. We report that one compound (Br-du) altered drastically embryogenesis, which argues in favor of this simple technique as a cheap first screening of chemicals or drugs to be used in a number of genotoxicology applications.

## Background

Sex is evolutionary beneficial due to genetic variation in the offspring. Meiotic recombination and allele complementation are two mechanisms inherent to sexual reproduction through which individuals adapt to the environment. Recombination will bring together advantageous alleles on the same chromosome that would be inherited as an assembled entity and new gene combinations might be selected for their fitness in a given environmental toxicology context. Moreover, genetic recombination at the meiosis stage is inherently linked to DNA repair mechanisms of damage in double-stranded DNA, which is usually lethal if not corrected [1,2]. Some workers have proposed that the genome in asexual reproduction accumulates deleterious mutations on single or double stranded DNA. In evolutionary biology, this is called the Muller's ratchet paradigm and this means that clonality compels genomes to be inherited as they are without the highly efficient meiosis recombination repair observed in sexuality [3]. We might predict that once the genomes in an asexual population bear one or more deleterious mutations, progenies are expected to die. The increased growth rate of clonal populations versus sexual populations usually observed in many bimodal species suggests that many unfit individuals in a clonal population are not a threat for the species [4,5].

Parthenogenetic insect species are not concerned by independent sorting of chromosomes that occurs through meiotic segregation and are probably free of chromosomal recombination events [6-8]. Therefore, they are logical valuable models to analyze the intertwined effects of epigenetic events and toxicological agents. We reasoned that asexual species like aphids might be an interesting model of clonality in order to investigate the genotoxicity of some compounds by bypassing the efficient check point of meiotic recombinatorial DNA repair. We thought that the genotoxicology of some compounds might be examined with this highly sensitive genome system if we accept that DNA repair mechanisms in clonal species are rudimentary and at this stage they are little known. Genotoxicity of compounds might be visible in asexual species but unnoticed and/or at sublevel detection in sexual species. Clonal models might be an alternative to investigate the chronic exposure of chemicals for which testing on mammal models turned out to be extremely difficult. Though, basic mechanisms and molecules in cell biology from yeast to mammals are conserved, toxicity processes are highly divergent between species and individuals within the same species. Nevertheless, the proposed method is designed to study the effects of compounds using the basic and conserved principles of cell biology. In this regard, it has been reported that at the DNA and chromosome level marine invertebrates express qualitatively similar types of induced chemical damage to that found in higher organisms (point mutations, strand breaks and chromosomal aberrations) [9].

The aphid *Acyrthociphon Pisum *is an example of a parthenogenetic insect generating distinct morphs [6,7]. They feed by sucking the intercellular liquid of leaves from specialized host plants, giving birth to larva which before adulthood will go through 4 moulting stages [6,7]. An advantage of the Aphid model is that they have multiple germaria from which embryos invaginate one behind the other and which are directly accessible after cuticle dissection. The germarium *plus *the maturing embryos (each one behind the other) constitutes an ovariole and six to nine of these ovarioles are present in a single parthenogenetic aphid [10].

It is assumed that plant respiration drives water and metabolites from roots to leaves. The concentration of metabolites and their distribution kinetics in the cellular compartment partly depends on their physicochemical properties. Therefore, amphoteric substances and appropriately hydrophobic/hydrophilic balanced molecules soluble in water should be good candidates for transport from the plant stem to the leaves. These chemicals if not taste deterrent will concentrate in aphids during the time they suck the phoem. To analyse toxicological effects in the aphids, three different types of soluble chemical bearing a bromo group which allowed their passage across the membrane were studied. The first was bromo-deoxyuridine (Brdu) and the two other molecules were Br-cAMP and Br-cGMP. Bromo-deoxyuridine (Brdu), an analog of thymidine passes through the plasmatic and nuclear membranes before getting incorporated into DNA during the replication process of cell division [11]. The advantage of using this molecule is that its incorporation can be monitored by immuno-histology techniques [12]. Br-cAMP and Br-cGMP are commonly used as permeable analogues of cAMP and cGMP and mostly used as non hydrolysable activators of protein kinase PKA and PKG [13-18]. These molecules, targeting different cellular compartments and mechanisms, were used as generic models to evaluate the efficiency and accuracy of the designed protocols.

We took advantage of the fact that aphids present three generations within the same parthenogenetic aphid (mother/embryo/embryo nascent in the old embryo) which constitutes a telescopic generation system [6]. The effects of chemicals were analyzed to determine at which level they induce deleterious effects. Therefore, these compounds acting on known cellular metabolic pathways were tested to address whether the experimental design was able to highlight chemical induced disorders in the first, second and/or third generations.

## Methods

### Maintenance and propagation of aphids

The 'Pea Aphid' *Acyrthosiphon pisum *belongs to the order of the Homoptera Family (Aphididae). They were maintained on *Vicia faba *plants in cages in an incubation room at 20°C ± 3°C and a photoperiodicity of 16/8 hours light/dark.

### Ingestion of chemicals by aphids

**Figure 1:**
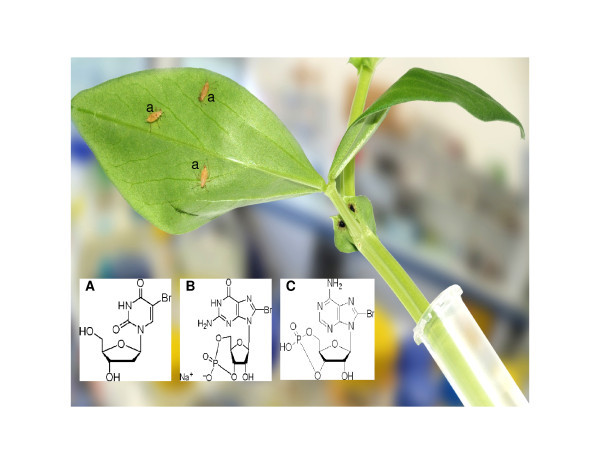
**Plant/aphid system designed for pharmacological testing**. A cut stem with two or three leaves of pea *Vicia Faba *was placed in a microfuge tube (1.5 ml) filled with either chemical (treated) (50 μM) or water (control). **A**: *Brdu *(bromo-deoxyuridine), **B**: *Br-cAMP*, **C**: *Br-cGMP*. Few aphids (a) were then placed on the leaves for 10 hours then after on a fresh plant for three days before further investigation.

We developed a simple technique which consisted of placing a razor cut stem of a pea leaf in a 1.5 ml microfuge tube filled with either water or the chemical under study [figure 1]. The added molecules migrate to the leaves through the plant vascular system by capillarity and diffusion in phloem sap. The aphid is able to rest immobile for hours on the plant leaf. Using it's stylet it can reach into the phloem and ingest large volumes of sap containing the added chemical substance. Adult aphids were placed for 10 hours on leaves in a microfuge tube containing the chemical under study or water as a control, then onto a fresh plant as described in figure 1. The animals were dissected 3 days after the chemical feeding step.

### Immunocytochemistry

Adult wingless/wing aphids were dissected with surgical forceps under a microscope (×10 magnification) and the germarium/ovariole structures were removed. The germarium/ovarioles were immediately immobilized in 5% glutaraldehyde in PBS for 3 minutes (in some experiments, alternative fixations with 1% glutaraldehyde or 70% ethanol were used). The germarium/ovarioles were then washed twice in PBS and incubated with 1% BSA in PBS at 4°C for one hour. The samples were then incubated with rabbit anti-HRP (horse radish peroxidase; Sigma) at a 1:10,000 dilution or anti Brdu (Sigma) in PBS at 4°C for overnight. Samples were then washed twice in PBS and incubated with a goat anti rabbit antibody conjugated to fluorescein isothiocyanate FITC (Sigma; 1:10,000) for 2 hours at 4°C.

### Demography analysis

Aphid larval development is characterized by four stages between birth and adulthood. Aphids at day 15 were placed on *Vicia faba *leaves for chemical absorption (Brdu, Br-cAMP, Br-cGMP) as described in the legend of figure 1. Ten hours after insects are moved to fresh plant leaves and the progeny was counted at day 12 according to their larva stage. Results are represented as a relative index determined by a control trial. The same experiment was carried out with the second generation progenies in the same conditions. Numbers are reported as a ratio of the number obtained with the control. The average of three experiments were statistically analysed by the *Student T *test with +/- SE (standard error) and a *p *value.

## Results

**Figure 2:**
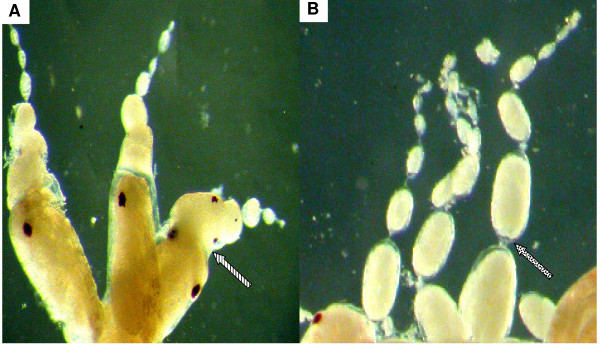
**Aphid ovarioles (germarium *plus *embryos at different stages) after genomic *Brdu *incorporation**. Aphids can concentrate chemicals in the hemolymph by the use of the drug delivery system described in figure 1. Animals were dissected with the aid of a microscope and the ovarioles were fixed in methanol 70% for four hours. **A**: ovarioles submitted to exposure of *Brdu*. We observe the malformation (fusion) of intermediate stage embryos (striped arrow) and the identical size of early embryos. **B**: control of ovarioles without chemicals. We see the well defined and successive stages of maturing embryos (spotted arrow) each one behind the other in their respective ovarioles.

**Figure 3:**
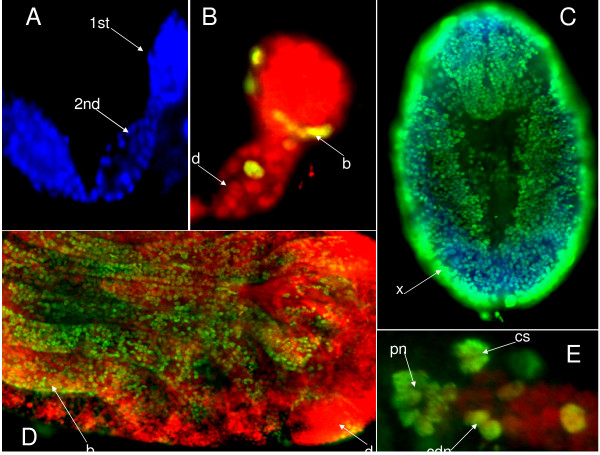
***Brdu *nuclei staining of embryos at different stages**. Aphids were submitted to plant/chemical feeding as described in figure 1. **A**: DAPI (4,6'-diamidino-2-phenylindole) blue fluorescent dye for staining nuclei reveals a germarium, first (1^st^)and second (2^nd^)embryo. **B**: Stained nuclei by DAPI (d) are represented in red. Green nuclei are *Brdu *(b) staining using an antibody FITC (Sigma Aldrich). We observe rare nuclei under replication. **C**: An early embryo showing numerous nuclei stained with anti *Brdu *FITC. We see the partially empty center and the external border enriched in giant nuclei (x) likely from polynucleated cells. **D**: old embryo before birth. Nuclei are stained with DAPI (red) (d) and the *Brdu *labelled nuclei (green) (b) are essentially present in legs, antennae and stylet. **E**: Representation of four types of cell division. We see replication in polynucleated cells (pn), chromosome segregation (cs), compact double nuclei (cdn) and nascent process of replication.

**Figure 4:**
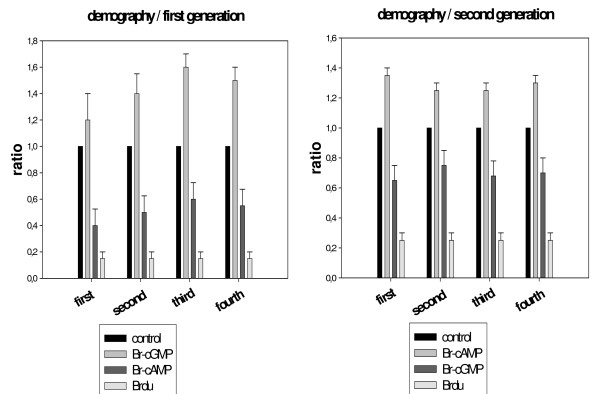
**Aphid drug absorption: demography effects on the first and second generation of progeny**. The plant leaf system was used to transfer chemicals into adult aphids (25 μM in 1.5 ml water) for 10 hours. Then the aphids were placed on a fresh plant. The progeny demography was counted for each larval stage at day 12. The second generation was also counted at day 12 due to the fact that a nascent embryo emerges in older embryos before birth (aphids are like "russian dolls" with three generations). Results are the mean+/-SE of three separated experiments. Br-cAMP and Br-cGMP *versus *control, p < 0,05 and Br-du versus control p < 0,005.

Ovarioles of treated aphids were microscopically examined after chemical ingestion [figure 1 and see method section]. Aphids which had ingested the chemical Br-du, revealed partially fused embryos [figure 2A], that had failed to dissociate from each other, as well as the presence of 4/5 early embryos in the same ovariole with the same size, denoting a blockage of the developmental process. The control [figure 2B] allowed us to examine in a simple/accessible way comparatively at which step the failure of the process occurred. In Br-cAMP, Br-cGMP treated aphids, microscopic examination revealed a less clear picture (data not shown). We then analyzed the incorporation of Br-du groups in the embryos. Embryo immuno staining showed distinct labelled individual cells and a group of labelled cells dividing intensively whereas other structures looked quiescent. The photographs reveal different stages of cell division and chromosome replication in polynucleated cells [figure 3]. Finally, using the three drugs demography studies were carried out. Due to that aphids are like "Russian dolls" presenting three generations in the same animal (the parthenogenetic mother/embryo^1^/a nascent embryo in older embryo^1^), the studies were conducted in the successive two generations. Br-du as expected presented a strong lethality effect followed by Br-cAMP. On the other hand, Br-cGMP increased the demography in the two generations, although results were consistently more marked in the first generation [figure 4].

## Discussion

Herein, we describe a simple and versatile technique to screen drugs and/or chemicals for pharmacological and/or toxicological purposes. By direct observation of the temporal order of embryogenesis in one single parthenogenetic mother in which all the intermediate forms co-exist, the proposed system might function as a map to pinpoint the precise timing where a drug induces a deleterious effect in a cell or alternatively manipulates phenotype outcome later in the adult. This method can be extended to study other processes affected by chemicals like the formation of the blastula (see Additional file 1) or the formation of gastrula (see Additional file 2). This biological system would provide also a fast and reliable method to examine events like formation of the neural plate and/or the central nervous system using an antibody against a neuronal insect marker [19] (see Additional file 3). Also it could be used in studies of segmentation genes which establish the segmented body plan of the embryo along the anterior-posterior axis like the Antennapedia group and bithorax group of homeotic genes [20-22]. Similarly, drugs that act on molecules (as *Wingless *protein) known to activate the adjacent rows of cells by binding to their cell surface receptor (like *Frizzled *protein) in order to specify anatomical structures [20-22], might be investigated. Finally, one could also envisage this technique being used in eco-toxicology studies to analyze the effects of compounds for pesticide properties in pest control and crop protection and also whether they have deleterious effects on other organisms than the one the chemical was produced for.

We have so far only studied three molecules and have seen adverse effects caused by all three. However, as stated in the introduction, amphoteric substances and appropriately hydrophobic/hydrophilic balanced molecules soluble in water should be good candidates. Therefore, one could presume this model would be limited as certain molecules may not pass through the plant vasculature to reach the aphid. This and other constraints would have to be further investigated in new studies by testing other molecules with different properties and functions. There are limits to all animal models used in toxicology studies at present. Recently it has been reported that toxicological trials have been carried out in several animal species and the type and range of effects between animal species were similar for only 60% of tested chemicals [23]. Secondly in the pharmaceutical industry there is a 20% failure of drug candidates due to side effects and toxicity which only becomes evident after they are administered to humans in clinical trials [23].

In conclusion, this simple "plant to animal feeding system" developed to study the biological effects of chemicals/drugs is much cheaper and less complicated than other models such as authorized mammal models. It can clearly be used to obtain an idea on the toxic effects of new chemicals or drugs on the early life stages of an embryo (a mix of differentiated cells *in vivo *compared to single clonal *in vitro *cell culture techniques). Although, the aphid embryo does not resemble at all a human embryo in any way, it is made up of a mix of differentiated cells. Any deleterious effects seen in aphid embryos would require supplemental studies in other systems to estimate and to predict harmful effects of chemicals on mammals. Also, of importance this method avoids the ethical problems of using larger animal models at the preliminary investigation phase of new chemical compounds for their genotoxicity.

## Competing interests

The authors declare that they have no competing interests.

## Authors' contributions

AD and AR conceived, designed and implemented the method. GE performed the fluorescence microscopy analyses. AR and TNL wrote the manuscript.

All authors read and approved the final manuscript.

## Supplementary Material

Additional File 1 **Movie of corazonin staining in geramarium**. This Movie shows a germarium and the first envaginated embryo labelled with anti corazonin antibody labelled with anti secondary antibody FITC (The gene for corazonin has not been found which might suggest cross-reactivity with other peptides). Click here for file

Additional File 2 **Movie of allatostatin staining of embryo**. This movie shows an embryo labelled with anti allatostatin and the secondary anti FITC antibody. Click here for file

Additional File 3 **Movie of HRP staining of embryo**. This movie shows an embryo labelled with anti HRP (neuronal marker) and the secondary anti FITC antibody. Click here for file
